# Recent Advances in Volatiles of Teas

**DOI:** 10.3390/molecules21030338

**Published:** 2016-03-11

**Authors:** Xin-Qiang Zheng, Qing-Sheng Li, Li-Ping Xiang, Yue-Rong Liang

**Affiliations:** 1 Tea Research Institute, Zhejiang University, # 866 Yuhangtang Road, Hangzhou 310058, China; xqzheng@zju.edu.cn (X.Q.Z.); qsli@zju.edu.cn (Q.S.L.); 2 Guizhou Tea and Tea Products Quality Supervision and Inspection Center, Zunyi 563100, China; gzzyzj_2009@vip.sina.com

**Keywords:** *Camellia sinensis*, aroma, sensory quality, green tea, oolong tea, black tea, white tea

## Abstract

Volatile compounds are important components of tea aroma, a key attribute of sensory quality. The present review examines the formation of aromatic volatiles of various kinds of teas and factors influencing the formation of tea volatiles, including tea cultivar, growing environment and agronomic practices, processing method and storage of tea. The determination of tea volatiles and the relationship of active-aroma volatiles with the sensory qualities of tea are also discussed in the present paper.

## 1. Introduction

Tea quality is defined by the appearance of dry tea, as well as the color, aroma and taste of tea liquor. Aroma, consisting of a group of volatile compounds, is an essential factor determining the quality of tea. Sensory properties in tea aromas were statistically correlated to the levels of volatile flavor components. Tea researchers paid great attention to tea volatile compounds to understand the aroma characteristics of various kinds of teas. More than 600 volatile compounds have been identified in tea aroma, so far [1]. The volatile composition of tea depends on the materials and processing methods used. There was a great difference in aromatic volatiles between various kinds of teas owing to the difference in fresh tea materials and processing methods. Some active-aroma volatiles significantly contribute to the flavor of tea because of their very low odor perception thresholds for human beings. A small change in the potent aromatic components may lead to a great difference in tea flavor. There is great potential to improve tea quality by improving tea cultivars, cultivation and/or processing techniques.

Recent advances in aromatic tea volatiles and the factors influencing the formation of tea volatiles, including tea cultivar, growing environment, cultivation practice, processing method and storage, determination of tea volatiles and the relationship between aromatic volatiles and the sensory quality of various kinds of tea were reviewed in the present paper.

## 2. Aromatic Volatile Compounds in Various Kinds of Teas

### 2.1. Formation of Tea Aromatic Volatiles

Volatile compounds in tea products can be formed through many pathways, such as the carotenoid derivatives pathway, the fatty acid derivatives pathway, the terpene derivatives pathway, the phenylpropanoid/benzenoid derivatives pathway, the glycoside hydrolysis pathway and the Maillard reaction pathway.

**Figure 1:**
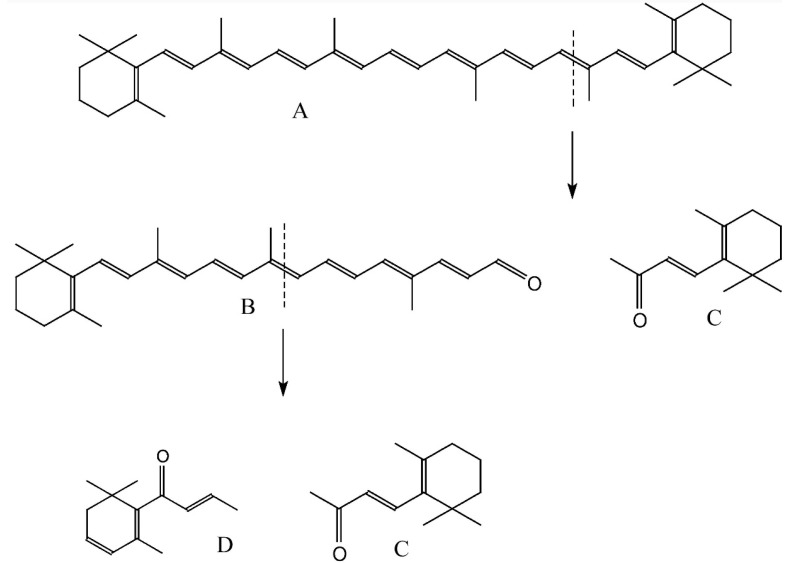
Oxidative degradation of β-carotene: (**A**) β-carotene; (**B**) 10’-apo-β-carotenal; (**C**) β-ionone; (**D**) β-damascone.

Carotenoids are important precursors of tea volatile compounds, especially the C_9_- to C_13_-aromas. Ionone and damascone are important C_13_-carotenoid-derived compounds (Figure 1) that constitute an essential aroma note in black tea. Testing in a model fermentation system consisting of β-carotene, tea catechins and crude soluble enzymes’ preparation extracted from fresh tea leaves showed that β-ionone was the major volatile product, and β-damascone was the minor product formed during the conversion process by oxidative degradation of β-carotene [2,3]. Concentrations of volatiles, including β-ionone, α-ionone, β-damascone and α-damascone, were significantly increased, and the volatile flavor compound (VFC) index was improved by supplementing carotenoid in tea leaves during black tea fermentation [4].

Lipids, especially fatty acids (FA), contributed greatly to the aroma and flavor volatiles in tea [5]. Glycolipids, which are rich in linolenic acid, are the most abundant lipids in fresh tea leaf. Neutral lipids, which are rich in linoleic, myristic, lauric, stearic, palmitic and oleic acids, are moderately abundant lipids. Phospholipids, which have a high proportion of linoleic, palmitic and oleic acids, are the least abundant lipids in tea [6]. During black tea processing, including withering, rolling and fermentation, the lipids are degraded to produce flavor volatiles by hydrolytic or oxidative action of enzymes on glycolipids and phospholipids [5]. The major fatty acid derivatives include alcohols, aldehydes and lactones. C_6_ and C_9_ alcohols and aldehydes are key contributors to the “fresh green” odor of tea. Methyl jasmonate, an important fatty acid derivative, is a major contributor to the jasmine-like aroma of oolong tea [7]. The aldehydes were synthesized via an intermediate, in which the primary reaction is the dioxygenation of an unsaturated fatty acid catalyzed by a lipoxygenase (LOX). The fatty acid-derived aldehydes could be further transformed to their corresponding alcohols by alcohol dehydrogenase [8].

**Figure 2:**
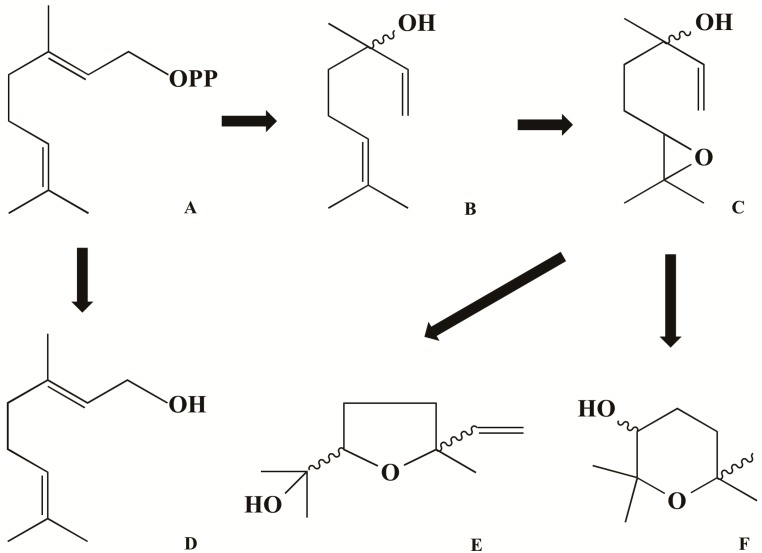
Formation of fatty acid-derived volatiles: (**A**) geranyl pyrophosphate; (**B**) linalool; (**C**) linalool-6,7-epoxide; (**D**) geraniol; (**E**) linalool oxide (puranoid); (**F**) linalool oxide (furanoid).

Geraniol, linalool and linalool oxides are important terpene derivatives in tea, especially in black tea. Geranyl pyrophosphate (GPP), a compound produced in the methylerythritol phosphate (MEP) pathway [9], is the precursor of the terpenoid derivatives geraniol and linalool by geraniol synthase and linalool synthase, respectively [10,11] (Figure 2). The linalool oxidation may be catalyzed by a non-specific enzyme [11]. Linalool is usually present in volatile free form in black tea. However, linalool oxides always occur in the bound forms of primeverosides [12].

Phenylalanine, a compound produced in the shikimic acid pathway, is the precursor of volatile phenylpropanoid/benzenoid derivatives [13]. The volatile phenylpropanoid/benzenoid derivatives are important contributors to the fruity and floral smells. Major phenylpropanoids in tea are phenylethanol and phenylacetaldehyde, and major benzenoids are benzyl alcohol and benzaldehyde [13]. The enzymes involving in these biosynthesis pathways in tea remain to be investigated.

Many tea volatile compounds are present in glycosidically-bound forms, which are more water soluble and more easily stored, but less volatile than their free aglycone counterparts. Benzyl β-d-glucopyranoside was the first volatile glycoside isolated from tea leaf [14]. It was later confirmed that the major glycosides in tea leaf were primeverosides and glucosides, among which primeverosides were predominant [15,16]. Biosynthesis of glycosides from free volatile compounds is catalyzed by glycosyltransferases [14]. Glycosidically-bound volatiles are located in the vacuoles, but glycosidases are present in the cell walls and cavity areas among cells [17]. The primeverosides and glucosides were hydrolyzed by the enzymes glucosidase and primeverosidase during tea processing, especially in the stage of rolling. Primeverosides are the major black tea volatile precursors, because they are abundant in fresh tea leaf, but almost disappear in the final tea products, whereas the glucosides are not substantially changed during tea processing [18]. Non-enzymatic hydrolysis of the glycosidically-bound volatiles takes place during tea processing. During the high temperature processing of ready-to-drink green tea and black tea, glycosidic precursors of damascenone are hydrolyzed [19]. 

The Maillard reaction is defined as a chemical reaction between reducing sugars and amino acids at high temperature, which produces brown pigments with caramel flavor. Roasted and pan-fired green teas contain high levels of Maillard reaction products, such as 1-ethyl-3,4-dehydropyrrolidone, pyrazines, pyrroles, pyrans and furans. Pyrazines and 1-ethyl-3,4-dehydropyrrolidone play an important role in developing a roasted flavor in both roasted and pan-fired green teas [20,21].

### 2.2. Variation of Aromatic Volatiles between Various Kinds of Tea

White tea, green tea, oolong tea, black tea and Pu-erh tea are major teas consumed in China. White tea is a minimally-processed tea. Fresh tea shoots with abundant leaf trichomes are withered under natural ambient conditions for a few days and then solar dried. The final product looks whitish owing to the dense grey white leaf trichomes covering the tea shoots. Green tea is an unfermented tea. During green tea processing, fresh leaves are heated in a steamer or a hot pan to inactivate the polyphenol oxidase so as to prevent tea polyphenols from oxidation. The fixed leaves are then rolled and dried. Green tea contains a high level of tea polyphenols. Black tea is a fully-fermented tea prepared by a processing procedure “withering—rolling—fermentation—drying”. Fresh leaves are withered in hot air and rolled in a cylindrical rolling machine or a machine called a CTC (crush, tearing and curling). The rolled leaves are fermented under controlled temperature and humidity, during which the tea polyphenols are oxidized by the polyphenol oxidase enzyme, resulting in the formation of red and orange tea pigments. Oolong tea is a semi-fermented tea whose processing procedure includes withering, shaking, rolling and drying. The aim of shaking processing is to partially bruise the leaf edge by hand touching or by a rotating drum. The polyphenols in the bruised parts of the leaves are oxidized. Pu-erh tea is a post-fermented tea, and its manufacture includes raw tea processing, fermentation and compressing. Fresh leaves are fixed and rolled as green tea. The rolled leaves are sun-dried and used as raw tea for post-fermentation. The raw tea is made damp and piled up for a few months, during which post-fermentation takes place by the action of mold. The fermented leaves are finally compressed into cakes or bricks.

Many aromatic compounds in fresh tea leaves are in the forms of non-volatile precursors or glycosides, which are liberated by glycosidase during tea processing [22]. The aromatic precursors and glycosidase enzymes vary with tea cultivars and processing methods. There are great variations of aromatic volatiles between various kinds of tea owing to the differences in tea cultivar and processing method. 

There were 133 aromatic volatiles formed during black tea processing, among which there were 48 carbonyls, 30 esters, 25 alcohols, 11 hydrocarbons, 3 phenols, 3 lactones and 13 volatiles of miscellaneous structures [23]. Volatile carbonyl compounds can be formed from the degradation of membrane lipids by enzymatic catalyzation. During black tea fermentation, catechins are oxidized. The oxidized catechins are strong oxidizing agents, which may oxidize other compounds, such as amino acids, carotenoids and unsaturated fatty acids, resulting in the formation of volatile compounds contributing to black tea aroma [24,25]. Linalool, methyl salicylate and C_6_-aldehydes increased appreciably during the rolling and fermentation of black tea, accompanying a decrease in unsaturated fatty acids. *Trans*-2-hexenal is increased with the loss of *cis*-3-hexenal during black tea withering and fermentation [6,25]. The aromatic volatiles are changed with the black tea fermentation, and so, the optimum fermentation time of black tea can be monitored using an electronic nose fitted with quartz crystal microbalance sensors [26,27].

There are two kinds of green tea, *i.e.*, steamed green tea made in Japan and pan-fired green tea made in China. All green teas contain high levels of indole pyridine, linalool, geraniol, benzyl alcohol, 2-phenyl-ethanol, 2-ethylhexanoic acid and maltol. Sulfinylbismethane and sulfonylbismethane are considered to be important volatiles contributing to the typical aroma of green tea. (*Z*)-1,5-octadien-3-one (metallic), 4-methoxy-2-methyl-2-butanethiol (meaty), 4-mercapto-4-methyl-2-pentanone (meaty), (*E*,*E*)-2,4-decadienal (fatty), β-damascenone (honey-like), β-damascone (honey-like), indole (animal-like) and (*Z*)-methyl jasmonate (floral) are the most important odorants of Japanese steamed green tea [28,29]. Geraniol (rose floral), linalool (sweet floral), linalool oxides (sweet floral), indole (animal-like), dihydroactinidiolide (sweet,), *cis*-jasmone (jasmine floral), 6-chloroindole (indole like), coumarin (sweet), methyl jasmonate (floral), trans-geranylacetone (fruity), phytol (sweet), 5,6-epoxy-β-ionone (rose floral) and phenylethyl alcohol (floral) are identified as various aroma-active compounds of green tea beverages prepared using steamed green tea [30,31]. However, Chinese roasted green teas contain high amounts of pyrazine (nutty), pyrrole (nutty), 4-hydroxy-2,5-dimethyl-3(2*H*)-furanone (sweet strawberry), 3-hydroxy-4,5-dimethyl-2(5*H*)-furanone (sweet strawberry), coumarin (sweet), vanillin (vanilla-like), geraniol (rose floral), (*E*)-isoeugenol (clove like) and 2-methoxyphenol (smoky). They have a high flavor dilution (FD) factor, which is defined as the highest dilution in which the tested odorant is perceived by trained panelists. (*E*)-isoeugenol (clove like) content is closely related to the green tea manufacturing process [32]. Moreover, 4-mercapto-4-methyl-2-pentanone, a roasted odorant contributing to the aroma of roasted green tea, increased with the increasing roasting temperature [33]. During the storage of green tea, 2-butyl-2-octenal (fruity) increased with time [34].

Oolong tea is a semi-fermented tea with a floral aroma. The major volatiles isolated from oolong tea were *cis*-jasmone (jasmine floral), β-ionone (rose floral), nerolidol (woody), jasmine-lactone (jasmine floral), methyl jasmonate (jasmine floral), indole (animal like), linalool and its derives (sweet floral) and geraniol (rose floral) [35,36]. These odorants are formed from precursors, such as (*S*)-linalyl β-primeveroside, *cis*- and *trans*-linalool 3,7-oxides 6-*O*-β-d-apiofuranosyl-β-glucopyranosides, (*Z*)-3-hexenyl β-d-glucopyranoside, methyl salicylate 6-*O*-β-d-xylopyranosyl-β-d-glucopyranoside and 8-hydroxygeranyl-β-primeveroside [12,37]. 

Microorganisms, such as fungus, are involved in the post-fermentation of Pu-erh tea processing, resulting in a moldy or musty smell. The major volatiles in Pu-erh tea included methoxyphenolic compounds, hydrocarbons and alcohol compounds, such as 1,2,3-trimethoxybenzene (storage musty), 1,2,4-trimethoxybenzene (storage musty), 2,6,10,14-tetramethyl-pentadecane (unknown), linalool and its oxides (sweet floral), α-terpineol (lilac), and phytol (sweet) [38]. The volatile composition of Pu-erh tea depends on the extraction method. Head space solid-phase microextraction (HS-SPME) was suitable for testing Pu-erh tea flavor [39,40].

White tea is a special tea processed using shoots with abundant leaf trichomes. There is abundant essential oil in the trichome joint by which the trichome is attached to the leaf lower epidermis [41], and so, white tea has high levels of volatiles, especially hexenal (green grassy odor) and (*E*)-hexenol (green grassy odor) [27]. 

The volatile composition is differentiated between various kinds of tea owing to the differences in cultivar and processing method, resulting in variation of aromatic characteristics. Table 1 lists the top ten volatiles in five kinds of tea. The different kinds of tea can be partially classified by cluster analysis using the index of individual catechins and volatile components [27].

## 3. Variation of Aromatic Volatiles between Tea Cultivars

There are great differences in aromatic volatiles between tea cultivars. For Japanese green tea cultivars, cultivar “Sayamakaori” had greater amounts of nerolidol, *cis*-jasmone and indole, but less linalool than cultivar “Yabukida” [42]. The *Camellia sinensis* var*. assamica* and its progenies, such as cultivars “Shizu-Inzatsu 131”, “Sofu” and “Fujikaori”, for Japanese green tea processing contain a specific volatile, methyl anthranilate [43]. The black tea made from var. *assamica* had much higher ratios of linalool, linalool oxides and methylsalcylate to total volatiles, but lower ratios of geraniol and 2-phenylethanol to total volatiles than hybrids of var. *assarnica ×* var. *sinensis* [44]. The differences in the volatile compounds between various types of tea depend on cultivar, growing environment and processing. Volatiles in fresh tea leaf are usually in the forms of glycosidically-bound precursors and are released by the action of hydrolases, such as β-primeverosidase, during tea processing [12,13,14,15,16,17,18,19]. Researchers are now attempting to screen tea cultivars with characteristically nice aromas according to the levels of glycosidically-bound precursors and the activities of hydrolase enzymes.

## 4. Effect of Environmental Conditions and Agronomic Practices on Aromatic Volatiles of Tea

The volatile flavor compounds (VFC) can be grouped into those imparting inferior aroma (Group I VFC), such as E-2-hexenal and hexanal with a green grassy, undesirable aroma, and those imparting a sweet flowery aroma (Group II VFC), such as geraniol and linalool with a floral aroma [45]. Environmental conditions have great impact on tea flavor. The total VFC, Group II VFC and flavor index, the ratio of Group II VFC to Group I VFC, in high grown black teas are higher than those in plain black teas [45]. The unsaturated fatty acids, the key precursors of volatiles contributing to tea aroma, increased with nitrogenous fertilizer rates [46]. However, excessively-fertilized green tea had high levels of high boiling point volatiles, such as β-ionone, 5,6-epoxy-β-ionone, dihydroactinidiolide and indole, resulting in a strong heavy aroma lacking briskness [47]. Plucking method and plucking round affect tea aroma. The coarse plucking standard results in a high level of Group I VFC [48]. Shear plucking and long plucking intervals produced coarser leaves than hand plucking and short plucking intervals, respectively, resulting in decreased level of flavor index [49] and the low sensory quality of tea. Infection by insects, such as tea leafhoppers and geometrids, induced the release of tea leaf volatiles [50], and the oolong tea made from leaves attacked by tea leafhopper had high levels of volatile compounds, including linalool-oxides, benzyl alcohol, 2-phenylethanol and 2,6-dimethylocta-3,7-diene-2,6-diol, leading to a unique aroma, like ripe fruit and honey [51].

## 5. Effect of Processing Methods on Aromatic Volatiles of Tea

Tea leaf volatile concentration changed during tea processing. During black tea processing, Group I VFC, which is dominated by *trans*-2-hexenal with a grassy flavor, increases rapidly during withering and fermentation and then decreases sharply during drying, while Group II VFC, which is dominated by linalool, phenylacetaldehyde and geraniol with flowery and sweet flavors, remains at a high level during the drying (Table 2), resulting in a rapid increase in the flavor index in the dry tea [52]. Though hard withering of black tea gave higher total VFC than normal withering, terpenoids decreased in hard withered leaves, resulting in a low ratio of terpenoids to non-terpenoids, which has a crucial effect on the flavor of black tea. It is considered that hard withering is deleterious to the sensory quality of black tea [53]. Changes of volatiles in green tea processing are quite different from those in black tea (Table 2).

Lipids and fatty acids are important precursors of aromatic volatiles, and the changes in lipid content/fatty acids are related to the volatiles produced in tea. Lipids in tea leaf include glycolipids, neutral lipids and phospholipids, which account for about 50%, 35% and 15% of the total lipids, respectively. There are considerable losses of lipids/fatty acids in the withering and drying process, while minor changes in lipid/fatty acid contents take place in the rolling and fermentation processes [6].

For oolong tea processing, leaf withering under direct sunlight or artificial light is accompanied soft hand-rolling every half an hour during the withering process [54]. Ultraviolet B with a wavelength from 280 nm to 320 nm was used as an artificial light to release the volatiles bound with glycosides by stimulating the enzyme activities of β-primeverosidase and β-glucosidase [55]. Some characteristic volatiles with potent odorants, such as linalool, furaneol and jasmine lactone, were produced during the manufacturing process [56].

## 6. Effect of Storage on Aromatic Volatiles of Tea

Levels of many aromatic volatiles, such as hexanal, *trans*-2-octenal, *trans-*2,4-heptadienal, *cis*-2,4-heptadienal, β-cyclocitral and β-ionone, are increased greatly during black tea storage. Changes in these volatiles can be used as indicators of tea aroma quality and as a means to distinguish teas that have been stored for various durations [57].

During oolong tea storage, long straight chain alcohols and acids are decomposed, while shorter-chain acids, their amide derivatives and many nitrogen-containing compounds are generated. The characteristic aromas of nitrogen-containing volatiles, including *n*-ethylsuccinimide, 2-acetylpyrrole, 2-formylpyrrole and 3-pyridinol, are regarded as typical constituents of old oolong tea [58]. During green tea storage at room temperature, levels of the volatiles 1-penten-3-ol, *cis*-2-penten-l-ol, *trans*-2-*cis*-4-heptadienal and *trans*-2-*trans*-4-heptadienal increased markedly, especially in low-grade tea and Ban-cha (an inexpensive Japanese green tea processed using coarse shoots, which contains more stems and twigs), while ionone derivatives, such α-ionone, β-ionone, β-cyclocitral, 5,6-epoxy-β-ionone and dihydroactinidiolide, which are formed by oxidation of carotenoids, increased slightly [59]. Storage of fresh tea leaf before green tea processing also affects the volatile compositions of tea. Green tea processed using leaves stored at low temperature (15 °C) has higher levels of volatiles with floral, fruity and sweet flavors than that stored at normal temperature (25 °C). During fresh leaf storage, indole increased initially, due to the transformation of anthranilic acid, and then gradually decreased by conversion from indole to oxygenated indoles [60].

## 7. Determination of Tea Volatiles

Determination of tea volatiles is very important in assessing tea quality, and it involves volatile extraction and instrumental analysis. The extraction methods can be categorized into solvent extraction, distillation sampling and headspace solids-phase microextraction (HS-SPME). There are great differences in volatile composition and concentration between various extraction methods. During solvent extraction, tea leaves are directly extracted in organic solvents, such as diethyl ether, pentane, hexane and ethyl acetate. Solvent extraction gives a complete profile of volatiles [31], but it always contains pigments and lipids, which interfere with the analytic instruments. Simultaneous distillation and extraction (SDE) is the most commonly-used distillation method for sampling tea volatiles [40]. SDE gives a high yield of volatiles, but its disadvantage is the presence of artifacts formed during the distillation process due to thermal reactions, oxidation and degradation [40]. To minimize the thermal reactions, steam distillation under reduced pressure (SDRP) at low temperature was developed to replace SDE [61]. HS-SPME is based on the adsorption and desorption of volatiles on SPME fibers coated with various adsorbents, such as 65-μm polydimethylsiloxane divinylbenzene. The SPME fiber was exposed to the headspace of the dry tea or tea liquor sample for about 60 min and then directly inserted into the GC/MS injector for desorption and testing [38]. The results of HS-SPME are closely related to sensory assessments, but are greatly influenced by sampling temperature and time. Therefore, it is crucial to maintain consistency in these factors.

GC/MS is usually used to analyze the volatile samples. Owing to the lack of the full set of authentic reference substances, the tested volatiles are commonly tentatively identified by the MS method and the concentration levels are always expressed as the ratio of the peak area of the detected volatile to that of an internal standard substance [55]. Therefore, the results from different sources are not comparable.

## 8. Effect of Aromatic Volatiles on the Sensory Quality of Tea

Sensory quality attributes include aroma, taste, liquor color, dry tea and infused leaf appearance, among which tea liquor taste and aroma are decisive factors. Water quality, which is related to indicators of pH, ion content and hardness, is a critical factor for the sensory quality of tea liquors, owing to the interactions between tea components and ions in water. Many studies showed that tea liquors extracted using purified waters by the treatments of distillation, reverse osmosis film or nanofiltration film gave better flavor than unprocessed water, such as chlorinated tap water [61]. 

Volatiles are key contributors to tea aroma. Each type of tea has its own typical aroma, because it has specific aroma-active volatiles. The major aroma-active volatiles in green tea, which usually has nutty-like or flowery aromas, include linalool, linalool oxides, geraniol, *cis*-jasmone, indole, coumarin, dihydroactinidiolide, methyl jasmonate, 6-chloroindole, 5,6-epoxy-β-ionone, *trans*-geranylacetone, phytol and phenylethyl alcohol [31], while the major aroma-active aroma volatiles in black tea, which usually has sweet and fruit like aromas, are 2-amylfuran, (*E/Z*)-2,6-nonadienal, 1-pentanol, epoxylinalool, (*Z*)-jasmone, 2-acetylpyrrole, farnesyl acetone, geranyl acetone, cadinol, cubenol and dihydroactinidiolide [62]. The key aroma-active volatiles detected in oolong tea, which usually smells floral, are *trans*-nerolidol, farnesene, hexyl hexanoate, indole and geraniol [63]; those detected in jasmine-scented tea with jasmine aroma are terpineol, nerol, jasmonate and methyl jasmonate [64]; and those detected in Pu-erh tea with a typical musty flavor are *n*-caproaldehyde, linalool oxide I,1,2,3-trimethoxybenzene and 1,2,4-trimethoxybenzene [39]. 

There are many studies focusing on integrating sensory assessments with volatile profiling, many of which are based on the quality-ranking methods [30,41,64,65,66,67], but few on the attribute-rating methods [31,68,69]. An attribute-rating study might be more important than quality ranking in determining the effect of volatiles on tea aroma. Average panelists can perceive several sensory attributes in tea liquors, but it is difficult for them to distinguish the differences between various scent attributes and even between scent attributes and taste attributes clearly [69]. This might be the reason why few attribute-rating studies were reported. It is necessary to perform further studies on this topic by trained and specialized sensory panels. 

## 9. Conclusions

The published literature shows that tea volatiles influence tea aroma through their composition and concentration, which are dependent on cultivar, environmental conditions, agronomic practices and processing methods. However, there is much more to be investigated in this research field. Firstly, the effects of glycosidically-bound volatiles (GBVs) on the formation of tea volatiles remain to be reconfirmed. It was reported recently that the enzymatic hydrolysis of GBVs was not involved in volatile formation [56], though many studies had emphasized its role [14,15,16,19,22,55]. Secondly, there is a long way to go for precise predicting of the sensory quality of tea by volatile testing, though many studies revealed the putative relationship of volatile profiles to sensory quality ranking [31,41,62,63,64]. The absolute concentrations of the volatiles were usually used to establish the predictive models. However, it is known that the sensory response of a volatile depends on its flavor value, which is defined as the ratio of absolute concentration to the sensory threshold of a compound, instead of absolute concentration level. For many volatiles in tea, the flavor values are not available owing to the lack of their sensory threshold data. Thirdly, it is necessary to develop efficient methods for extracting tea volatiles. SDE [41,64] and HS-SPME [63] are usually used to collect tea volatiles. SDE gives a high yield of the extraction of volatiles, but it often causes the emergence of artifacts owing to thermal reactions, degradation or oxidation during the long time distillation [65,66]. HS-SPME can avoid artifact formation, but its results are much lower than SDE [63]. The volatiles obtained by HS-SPME are not enough for the structural analysis of unknown compounds [63,67].

## Figures and Tables

**Table 1:** Top ten volatiles in various types of tea [27] ^1^.

No.	White Tea	Green Tea	Oolong Tea	Black Tea	Pu-erh Tea
1	Hexanal	(*E*)-Geraniol	(*E*)-Nerolidol	(*E*)-2-Hexenal	1,2,3-Trimethoxybenzene
2	(*E*)-2-Hexenal	Linalool	Indole	Hexanal	Linalool oxide II (cis, Furanoid)
3	Linalool oxide II (cis, Furanoid)	(*E*)-Nerolidol	Linalool	(*E*)-Geraniol	Hexanal
4	Linalool	(*Z*)-Hexanoic acid 3-hexenyl ester	Benzeneacetaldehyde	Linalool	Linalool oxide I (trans, Furanoid)
5	(*E*)-Geraniol	Nonanal	Phytol	Linalool oxide II (cis, Furanoid)	Benzaldehyde
6	Phenylethyl alcohol	3,7-Dimethyl-1,5,7-Octatrien-3-ol	α-Farnesene	Benzeneacetaldehyde	Benzeneacetaldehyde
7	Benzaldehyde	(*E*)-2-Hexenal	Linalool oxide I (trans, Furanoid)	Linalool oxide I (trans, Furanoid)	a-Terpineol
8	Linalool oxide I (trans, Furanoid)	Phytol	3,7-Dimethyl-1,5,7-Octatrien-3-ol	3,7-Dimethyl-1,5,7-Octatrien-3-ol	β-Ionone
9	Benzeneacetaldehyde	Heptanal	Benzyl nitrile	Benzaldehyde	Cedrol
10	(*Z*)-3-Hexen-1-ol	(*Z*)-Jasmone	Hexanal	Methyl salicylate	Linalool

^1^ Extraction method: simultaneous distillation and extraction (SDE); identification method: GC/MS tentative identification; quantitative analysis: ratio of tested compound peak area to that of the internal standard (1 mL of 5% (*v*/*v*) ethyl caproate). Sample number: white tea = 8, green tea = 21, oolong tea = 27, black tea = 15, Pu-erh tea = 10. The top ten volatiles were screened based on concentration.

**Table 2:** Effect of processing on the concentration of partial volatiles in black tea and green tea ^1^.

Processing Stage ^2^		A	B	C
*Trans-*2-hexenal	Green tea	52.619 ± 3.284	0.482 ± 0.139	0.205 ± 0.111
	Black tea	52.619 ± 3.284	60.906 ± 4.969	14.261 ± 0.235
Geraniol	Green tea	13.997 ± 2.845	0.982 ± 0.039	0.844 ± 0.05
	Black tea	13.997 ± 2.845	14.885 ± 0.425	15.05 ± 0.297

^1^ Plant material: shoots with one leaf and a bud picked from bushes of *Camellia sinensis cv. Zhenong-902*. Extraction method: headspace solids-phase microextraction (HS-SPME); identification method: GC/MS tentative identification; quantitative analysis: ratio of tested compound peak area to that of the internal standard (1 mL of 5% (*v*/*v*) ethyl caproate); ^2^ Stage A: fresh tea leaf; B: steaming fixed leaf for green tea and fermented leaf for black tea; C: dried tea.
